# Solitary thyroid metastasis from colon cancer: a rare case report

**DOI:** 10.3332/ecancer.2016.696

**Published:** 2016-11-24

**Authors:** L Nicosia, S Alessi, M Proh, E Grosso, M Ansarin, A Vingiani, E Pisa, E De Fiori

**Affiliations:** 1 Scuola di Specializzazione in Radiodiagnostica, Università degli Studi di Milano, Milano, Italy; 2 Divisione di Radiologia, Istituto Europeo di Oncologia, Milano, Italy; 3 Divisione di Otorinolaringoiatria e Chirurgia Cervico Facciale Istituto Europeo di Oncologia, Milano, Italy; 4 Divisione di Anatomia Patologica, Istituto Europeo di Oncologia, Milano, Italy

**Keywords:** thyroid metastasis, radiological follow-up, ultrasound imaging, fine needle aspiration (FNA)

## Abstract

Malignant metastases to the thyroid are rare and are even rarer from a colorectal primary. As these metastases are often asymptomatic, they are usually discovered incidentally on imaging performed as follow-up for the primary tumour. In this report, we present a case of metastatic sigmoid adenocarcinoma to the thyroid diagnosed and treated at our institution.

## Summary

The diagnosis of malignant metastases to the thyroid is rare: it is reported in only 1.4–3% of all patients who undergo surgery for suspected thyroid carcinoma [1, 2]; however, the actual incidence may be higher as they have been reported in 1–24% of cases in autopsy studies of cancer patients [1, 3]. In a review by Fadare *et al*, it was estimated that less than 100 cases of this phenomenon have been reported over the past century [3].

Kidney, breast, and lung tumours are considered the most frequent sources of metastases to the thyroid [4]. Metastasis from colon cancer is clinically rare, representing about 14% of all metastases to the thyroid [5–8]. Among the cases of thyroid metastases from colorectal cancer reported in the literature in the last five decades, only three cases were described as solitary thyroid metastasis without any other visceral metastases, suggesting that they usually occur in association with metastases to other sites [9].

Metastases to the thyroid rarely produce symptoms [2]. In fact, most of these metastases are detected incidentally during clinical and radiological follow-up in patients with a different primary carcinoma [10, 11].

In this report, we present a case of thyroid metastasis discovered incidentally during the follow-up of a patient with colorectal carcinoma, who had previously undergone surgical resection.

## Clinical presentation

A 75-year-old gentleman who had undergone sigmoid colectomy for adenocarcinoma of the sigmoid colon (pT3 N0 G2) in 2009 was being followed up with routine computed tomography (CT) scans.

In 2013, the patient was diagnosed with carcinoma of the prostate and received treatment with radiotherapy in 2014.

Follow-up CT in June 2013 also demonstrated metastatic left pulmonary nodules, and the patient subsequently underwent lobectomy of the left upper lobe of lung. Histological analysis confirmed metastatic adenocarcinoma of the colon (CDX2+). Between November 2013 and March 2014, the patient received four cycles of chemotherapy with Capecitabine.

Follow-up CT scan in September 2015 revealed the presence of a previously undiagnosed nodule in the thyroid gland. The lesion was investigated with positron emission tomography (PET) and ultrasound (US), before proceeding to fine-needle aspiration (FNA). Cytological analysis of the sample from the FNA led to the diagnosis of thyroid metastasis (TIR5) from adenocarcinoma of the sigmoid colon (CDX2+).

The patient denied dysphagia, dysphonia and aspiration. Clinically, palpation revealed a nodule in the right thyroid lobe but no enlarged cervical nodes.

Nasoendoscopy revealed normal vocal cord mobility and glottic respiratory space.

## Investigations/imaging findings

**Figure 1.**
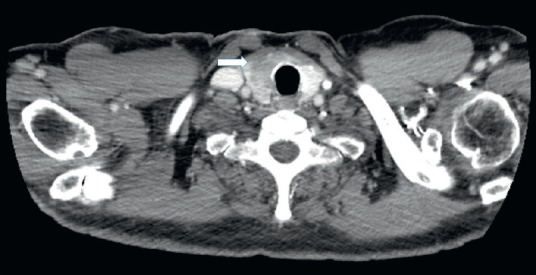
Axial CT scan after injection of contrast agent: 19 mm subtly hypodense nodule in the right thyroid lobe.

CT scan of September 2015 revealed a 19 mm subtly hypodense nodule in the right thyroid lobe (Figure 1).

**Figure 2.**
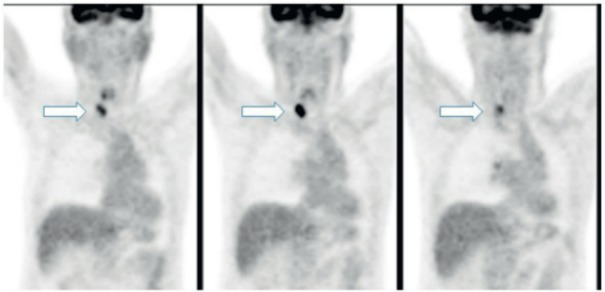
PET-CT: marked uptake in the right lobe of the thyroid gland.

The subsequent PET-CT with FDG of November 2015 demonstrated marked uptake in the right thyroid lobe, thus confirming the suspicion raised by the CT findings (Figure 2).

**Figure 3.**
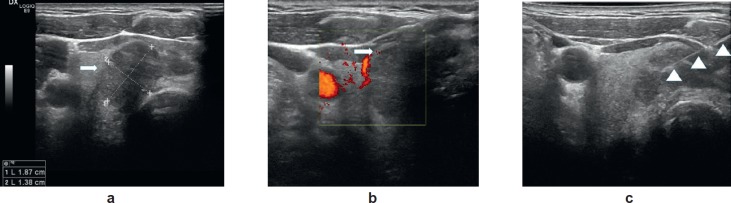
(a) Axial ultrasound image (6–13 MHz probe): a solid nodule measuring 19 × 14mm in the right thyroid lobe characterised by an incomplete peripheral hypoechoic halo and iso-, hypo-echogenic echotexture. (b) Axial ultrasound image with power Doppler: the absence of internal vascularity within the nodule (white arrow). (c) Fine-needle aspiration of the nodule (FNA); the needle (arrowheads) can be seen crossing the target.

The patient then underwent investigation by US (6–13 MHz high-resolution linear probe) that confirmed the presence of a taller than wide (19 × 14 mm), solid nodule in the right thyroid lobe. This nodule had an incomplete peripheral hypoechoic halo with iso-, hypo-echogenic internal echotexture and demonstrated absent internal vascularity on Doppler interrogation (Figures 3a and b).

**Figures 4.**
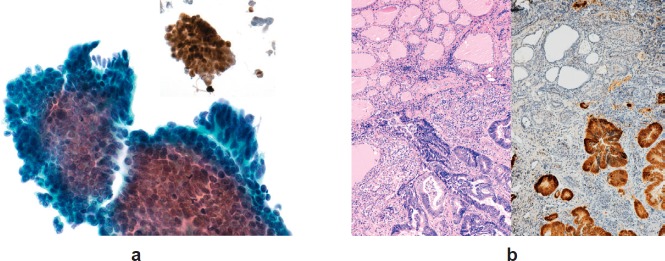
(a and b) Columnar cancer cells with dark, large, elongated, palisading nuclei on a background of necrotic debris. Immunohistochemistry was strongly positive for cytocheratin 20.

The patient was referred for FNA of the lesion, the cytological results of which were suggestive of intrathyroid metastasis from a colonic primary (TIR 5) (Figure 3c). This diagnosis was suggested by the recognition of columnar cancer cells with dark, large, elongated, palisading nuclei on a background of necrotic debris. Immunohistochemistry was strongly positive for cytocheratin 20, a marker of colon carcinoma (Figures 4a and b).

## Treatment

A multidisciplinary approach to treatment was taken and involved the discussion of the case at a multidisciplinary meeting in which radiologists, head and neck surgeons, general surgeons, radiotherapists, oncologists and endocrinologists participated.

In March 2016, the patient underwent right hemithyroidectomy preserving the parathyroid glands and the recurrent laryngeal nerve. Histology confirmed thyroid metastasis from adenocarcinoma of colonic origin. Immunophenotype of the neoplastic population was positive for CDX-2 and negative for TTF-1 and PSA.

The patient was discharged from the hospital with no indication for radiotherapy or chemotherapy. He is currently undergoing regular clinical and radiological follow-up.

## Discussion

Metastasis of malignant tumours to the thyroid gland are uncommon and they have been mainly observed in renal, breast, and lung carcinoma. Cases of metastases from colon rectal cancer, are extremely rare [12, 13]. Typically, metastases to the thyroid are detected as new manifestations of a known primary tumour; however, the primary tumour is sometimes diagnosed following the discovery of metastasis [3].

There are two theories that attempt to explain why metastases to the thyroid are so rare: the first suggests that the rich arterial supply and high vascularity of the thyroid gland prevents tumour cells from remaining fixed in the gland [14, 15]. In the second hypothesis, the high oxygen saturation and high iodine content in the thyroid are credited with preventing the growth of tumour cells from other organs [14, 16].

Thyroid metastases are more frequent in abnormal thyroid glands and are most commonly found concomitantly with goiters or follicular adenomas because of the decreased arterial blood flow, low oxygen level or low iodine content. Hypothyroidism and thyrotoxicosis can occur due to the infiltration of the gland by the malignant mass.

As in our reported case, thyroid metastases can present many years after initial diagnosis, making diagnosis even more difficult [1]. The colorectal metastasis to the thyroid was diagnosed 6 years after surgical resection of the colorectal primary and the patient had no symptoms related to thyroid function. The interval of more than 5 years is long enough to distract the physician’s attention from the previously treated malignant disease. Although a thyroid nodule is an extremely common clinical finding, the new appearance of a thyroid nodule in patients with previous malignant disease warrants prompt cytological evaluation to rule out the possibility of newly developed metastatic disease.

A thyroid nodule arising in a patient with a history of malignancy must be considered to be metastasis until proven otherwise. Fine-needle aspiration (FNA) with cytological evaluation reliably diagnoses primary thyroid neoplasms; this rule also extends to secondary malignancies. Notably, the number of cases with metastatic carcinoma to the thyroid seems to have increased [11]; this increase may be related to a concomitant increase in the number of FNA studies being performed.

Management of thyroid metastases should depend on the individual case. There is no clear consensus; however, some previous studies recommend a thyroid lobectomy and/or isthmectomy in the case of solitary thyroid metastasis and a total thyroidectomy in the case of bilateral metastases. Survival results of thyroidectomy depend on the primary tumour [17, 18].

Radiotherapy and chemotherapy are often reserved as palliative care for patients with widespread disease or in those with comorbidities that preclude surgical intervention [19]. The prognosis varies in relation to the grade of malignancy of the primary lesion; in general, renal cell carcinoma is of low grade malignancy, while lung and gastrointestinal carcinoma are often associated with spread to other organs with unfavourable prognosis [14].

The possibility of tumour metastases to the thyroid gland, although very rare, should be kept in mind when treating patients with a history of cancer elsewhere, especially in the case of a rapidly growing thyroid mass, dysphonia, and cough [20]. As in our case report, the discovery of the thyroid metastasis is often an incidental finding during follow-up imaging.

## Conclusion

We presented a rare case of thyroid metastasis from sigmoid carcinoma, a possibility that should be kept in mind whenever thyroid changes, clinical or radiological, are present in a patient with history of carcinoma elsewhere. In any patient with a known history of previous carcinoma, the appearance of a new thyroid mass should be regarded as being potentially metastatic. A thorough investigation for metastases to other organs should also be performed.
